# Counting Conditions on Newborn Bloodspot Screening Panels in Australia and New Zealand

**DOI:** 10.3390/ijns10030047

**Published:** 2024-07-05

**Authors:** Natasha Heather, Ronda F. Greaves, Kaustuv Bhattacharya, Lawrence Greed, James Pitt, Carol Wai-Kwan Siu, Mark de Hora, Ricky Price, Enzo Ranieri, Tiffany Wotton, Dianne Webster

**Affiliations:** 1 National Newborn Screening Laboratory, LabPlus, Health New Zealand Te Whatu Ora, Auckland 1023, New Zealand; 2 Liggins Institute, University of Auckland, Auckland 1023, New Zealand; 3 Victorian Clinical Genetics Services, Murdoch Children’s Research Institute, Parkville 3052, Australia; 4 Department of Paediatrics, University of Melbourne, Parkville 3052, Australia; 5 Discipline of Paediatrics and Child Health, School of Clinical Medicine, UNSW Medicine and Health, Sydney 2035, Australia; 6 Western Sydney Genetics Program, Children’s Hospital at Westmead, Sydney 2145, Australia; 7 Faculty of Medicine and Health Science, University of Sydney, Sydney 2006, Australia; 8 Western Australia Newborn Screening Program, Department of Clinical Biochemistry, PathWest Laboratory Medicine WA, Perth 6008, Australia; 9 Newborn Screening Laboratory, Department of Biochemical Genetics, Genetics & Molecular Pathology Directorate, SA Pathology, Women’s & Children’s Hospital, North Adelaide 5006, Australia; 10 Chemical Pathology, Pathology Queensland, Herston 4029, Australia; ricky.w.price@gmail.com; 11 New South Wales Newborn Screening Programme, The Children’s Hospital at Westmead, Sydney 2145, Australia

**Keywords:** newborn screening, bloodspot screening, counting conditions, screening panel, harmonization

## Abstract

A greater number of screened conditions is often considered to equate to better screening, whereas it may be due to conditions being counted differently. This manuscript describes a harmonised Australasian approach to listing target conditions found on bloodspot screening panels. Operational definitions for target disorders and incidental findings were developed and applied to disorder lists. A gap analysis was performed between five, state-based Australian newborn screening programme disorder lists and the single national New Zealand and state-level Californian versions. Screening panels were found to be broadly similar. Gap analysis with Californian data reflected differences in jurisdictional approval (for example, haemoglobinopathies and lysosomal disorders not being recommended in Australasia). Differences amongst Australasian panels reflected varied the timeframes recommended in order to implement newly approved disorders, as well as decisions to remove previously screened disorders. A harmonised approach to disorder counting is essential to performing valid comparisons of newborn bloodspot screening panels.

## 1. Introduction

**Figure 1:**
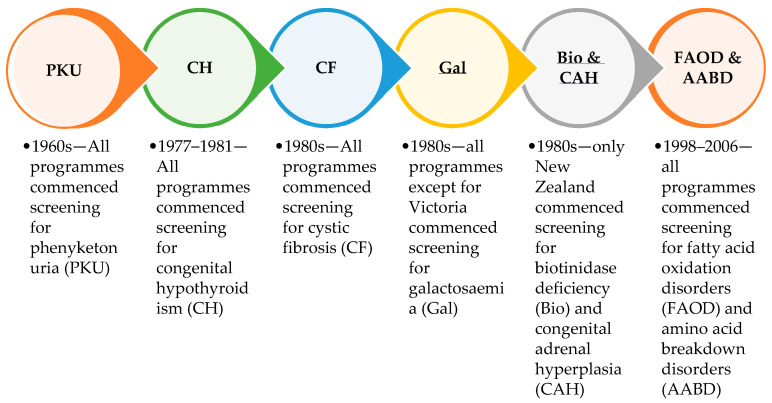
Conditions introduced across the six Australasian screening programme laboratories in the first 50 years of newborn bloodspot screening.

Newborn bloodspot screening (NBS) was established throughout Australia and New Zealand, collectively called Australasia, in the late 1960s. The five Australian screening laboratories are funded individually by state and territory governments, and there is a single national screening laboratory in Auckland, New Zealand. The Australian screening laboratories are located in Sydney (New South Wales), Brisbane (Queensland), Adelaide (South Australia), Melbourne (Victoria), and Perth (Western Australia). For states and territories with no screening laboratory, bloodspot samples are sent interstate for testing. Historical differences in funding, geographical considerations, and resources have influenced the addition of screened disorders and led to variations between programmes (Figure 1). 

Phenylketonuria (PKU) was the initial disorder to be screened in newborns, and was introduced throughout Australia and New Zealand in the late 1960s. Congenital hypothyroidism (CH) was added in the three Australian screening laboratories (Sydney, Melbourne and Adelaide) in 1977, in Auckland in 1978, in Brisbane in 1980, and in Perth in 1981. Cystic fibrosis (CF) screening began as a research pilot in New Zealand in 1979 before being added to the national screening programme in 1986. CF screening was introduced into the Sydney laboratory in 1981, and more slowly throughout Australia by 1999. Galactosaemia (Gal) screening was introduced to all Australasian screening laboratories in the 1980s, except for Victoria, where clinical diagnosis was deemed sufficient. Also, in the 1980s congenital adrenal hyperplasia (CAH) and biotinidase deficiency (Bio) were added to the New Zealand-based but not Australian screening panels. Fatty acid oxidation and amino acid breakdown disorders (FAOD and AABD), measured by tandem mass spectrometry of amino acids and acylcarnitines, which is commonly referred to as expanded NBS, were first introduced into the Sydney screening laboratory in 1998, and spread throughout Australia and New Zealand by 2006. Around 25 different fatty acid oxidation disorders and organic acidaemias were added to Australasian screening panels, with some variation between programmes. Individual disorders were not always considered in detail, as there was little information available about many of the rarer conditions.

The Human Genetics Society of Australasia (HGSA) formed an expert committee on NBS in 1979, incorporating members from screening laboratories and treating physicians. The HGSA is a peak professional body which has been setting standards for newborn screening since 1991, independently of New Zealand and Australian governments. The HGSA NBS committee reviews and updates policy on NBS in Australasia, with the aim of aligning programmes. More recently (2018), an Australian National Policy Framework was developed in order to guide future growth [1]. This significant document was developed by a working group consisting of scientific and clinical specialists in NBS, bioethicists, policy experts, midwifery, and parent consumer representatives. The policy framework addressed NBS implementation, quality and safety, program monitoring, evaluation, and also the decision-making process for disorders. This latter policy area detailed the pathway for adding or removing disorders from the screening panel, and the decision-making criteria. CAH screening was the first condition recommended in 2018 under this framework and was implemented across all five Australian jurisdictions by the end of 2022. Similarly, severe combined immunodeficiency (SCID) and spinal muscular atrophy (SMA) were recommended for introduction in 2020 [2,3,4]. By comparison, in New Zealand, CAH screening commenced in the 1980s, SCID screening commenced in 2017, and SMA screening has been approved to commence in 2024 [5,6].

In 2022, non-professional advocacy comparing Californian newborn screening to that in Australia indicated that Australia had not updated its screening programme since the 1980s and screened 25 screened conditions as opposed to “80 in California” [7]. The implication was that the greater number of screened conditions equated to better screening. However, actual screening outcomes need to be determined to judge the performance of screening rather than counting the number of detectable disorders. High-quality screening programmes have a rigorous process for selection of disorders with interventions that can be delivered with good screening performance as per the Wilson and Junger World Health Organisation criteria [8]. Some centres in Australia have performed tandem mass spectrometry screening for 25 years, being the first publicly funded centres worldwide to do so [9,10]. After initial implementation, the HGSA NBS committee did not recommend screening for short-chain acyl-CoA dehydrogenase (SCAD) deficiency as it is considered benign [9]. Other conditions, such as ornithine transcarbamylase deficiency (OTCD) and non-ketotic hyperglycinaemia (NKH), are also not screened for as classical cases need urgent intervention before the screening results become available on day 7–10 of life, as well as due to the lack of a sensitive marker for OTCD [10,11]. 

Furthermore, there is a lack of uniformity in condition counting within the international community, which leads to difficulty in comparing screening panels [12]. Some of the difficulties relate to primary analytes, which may indicate a range of genetic conditions. For example, elevation in propionylcarnitine (C3 carnitine) may be due to variants in multiple genes including MMUT, MMAA, MMAB, MMADHC and MCEE [13]. This can lead to ambiguity in nomenclature and regarding the extent to which genetic variants and conditions should be grouped or listed individually. Other issues include whether the list of disorders is restricted to conditions which have been approved as meeting jurisdictional screening criteria or more broadly refers to those which may (sometimes) be detectable [12]. 

The HGSA NBS committee has coordinated screening in Australasia for decades, with experience in harmonising several approaches. The committee undertook a collaborative process to categorise and list bloodspot conditions in a standardised manner. The aim was to produce a list of target disorders comparable across Australasia to provide a resource for government policy makers.

## 2. Materials and Methods

### 2.1. NBS Programmes

The newborn screening committee of the HGSA represents the five state-based Australian and the single New Zealand national NBS programme. Together, these six programs test approximately 350,000 newborns annually. Between July 2022 and February 2023, the committee met regularly for discussions on disorder counting. 

### 2.2. Disorder Lists

As it had been politically used as a comparison, the HGSA NBS committee utilised the disorder list from the Californian Department of Public Health website as a starting point in developing a comparable Australasian list of screened disorders [14]. As of October 2020, the Californian screening website listed 35 core conditions (33 of which were screened on bloodspots) from the recommended uniform screening panel (RUSP). RUSP core conditions are recommended for screening by states as part of their state universal newborn screening programmes by the US Secretary of the Department of Health and Human services [15]. Secondary disorders, defined as disorders which can be detected in the differential diagnosis of a core disorder, were also listed as of September 2018. These were divided into lists of RUSP secondary conditions (22 conditions across five categories), with other secondary conditions which can be detected by the Californian programme (12 conditions) and other secondary haemoglobinopathies which can be detected by the Californian programme (17 conditions across four categories of haemoglobinopathy).

### 2.3. Definitions of Target Disorder and Incidental Findings

The concepts of screening targets and incidental findings were discussed, and operational definitions were developed. These were applied to existing disorder lists from the six Australasian screening programmes, with the intent of reaching a consensus where possible. Core disorders from the Californian list were similarly discussed within the Australasian context and categorised as being target conditions, incidental findings, or as not currently screened within Australasia.

### 2.4. Gap Analysis

Target screened disorders were compared both within Australasia and in relation to the Californian list of core conditions, and gap analysis was provided. Critical congenital heart disease and hearing loss were excluded as not being screened on bloodspots, leaving a total of 33 core conditions from the Californian list. 

## 3. Results

### 3.1. Definitions of Target Disorder and Incidental Findings

Initial discussions were complicated by the concepts of “screening targets” and “incidental findings”. The operational definitions developed collaboratively by HGSA NBS committee members are summarised in Table 1. A target disorder was understood as being required to meet the jurisdictional interpretation of the Wilson and Junger screening criteria [1,8,9,16]. Conditions which had been formally approved in an Australasian jurisdiction were therefore included as target conditions; however, not all disorders had been through this process. For example, it was noted that many disorders had been added as a group at the onset of tandem mass spectrometric analysis of acylcarnitines, amino acids and other metabolites (MSMS screening) without being assessed individually. This definition also required an intent to screen with maximum sensitivity and specificity, although low sensitivity could be accepted if it could be determined across the population and was appropriately balanced with (high) specificity and clinical utility. Prior to this process, these conditions were classified by the HGSA as category 1 disorders.

Conversely, incidental findings were understood as having the same marker metabolite as a target condition, but not meeting the screening criteria. This category therefore included conditions where screening sensitivity could not be determined, or where the benefit of early identification was unclear. Furthermore, it was acknowledged that it may not be possible to distinguish an incidental finding from a target disorder in the screening laboratory without confirmatory follow-up testing. In the process of screening for target conditions, all Australasian screening laboratories recognised the detection of incidental findings. It was understood that the sensitivity in detecting these would vary depending on the technology, method, and cutoffs utilised when screening for target disorders. Prior to this process, these had been classified as category 2 disorders. The term “incidental finding” was preferred as this was not the primary reason for using a particular analyte for screening purposes. 

### 3.2. Disorder Lists

The summary list of bloodspot conditions was published on the HGSA website as a policy document in February 2023, and further updated in March 2024 [17]. This document provides a table of target disorders screened by jurisdiction, alongside a separate list of (possible) incidental findings. This summary document was shared with Australian health funders and policy makers in 2023 to assist with national policy in order to harmonise disorder panels.

### 3.3. Gap Analysis

Table 2 compares Australasian screened disorders to those in California. Gaps included disorders which were not approved in Australasian jurisdictions. As of February 2023, no lysosomal storage disorders were under active consideration in Australia or New Zealand, X-linked adrenoleukodystrophy was under active consideration in both countries, and sickle cell disease was under active consideration in Australia only.

Other gaps between the Californian and Australasian panels included recently approved disorders, where implementation was intended to be performed but not yet complete. For example, SCID and SMA screening had been recommended throughout Australia in 2020, but implementation was dependent on state-based funding and resources and required individual laboratories to prepare successful business cases. The Sydney laboratory was able to implement these rapidly measures following initial pilots, whilst other Australian screening programmes remained in the process of obtaining the necessary funding and instrumentation. The New Zealand screening laboratory included SCID as a screening target. However, this did not include SMA as this was still under active consideration by the New Zealand Ministry of Health in February 2023.

Some differences amongst Australasian programmes were seen due to the historical decisions made for screened disorders. For example, galactosaemias (classic and other) were target disorders by all Australasian screening laboratories, except that in Melbourne [11,18]. This was because treating physicians in the 1980s elected to detect these cases clinically rather than through screening. Similarly, CAH and biotinidase deficiency screening were introduced in New Zealand, but not Australia in the 1980s.

Tyrosinaemia type I was considered to be a target condition by the New Zealand screening laboratory but was an incidental finding in the five Australian screening laboratories, which used tyrosine as a first-tier marker. The New Zealand laboratory suspended screening for tyrosinaemia type I in 2017 due to poor screening performances when using tyrosine as the screening marker [19], and re-introduced this in 2022 following the development of a method for analysing succinylacetone as a more sensitive and specific marker. At the time of discussion, none of the Australian laboratories had a first-tier succinylacetone method available as validated in-house IVDs necessitated separate extraction and MSMS analysis and were considered too resource-intensive. All laboratories recognised that the tyrosine cutoffs in use were too high to reliably detect disease, so it was not considered as meeting screening target criteria for Australian programmes. 

Other differences reflected disorders which had been formally removed from the New Zealand screening panel, such as carnitine uptake disorder and 3 methylcrotonyl-CoA carboxylase deficiency (3-MCC). Carnitine uptake disorder is a screened condition throughout Australia, but has not been detected New Zealand since 2016 due to the harm of screening likely out-weighing benefits [20]. Screening for 3-MCC was similarly discontinued in 2014, due to similar concerns about unclear screening benefits [21]. Following this decision, hydroxyisovalerylcarnitine (C5OH) levels were no longer utilised as a screening biomarker, so that other rare conditions screened using C5OH were also no longer detectable. Conversely, the Australian laboratories continued to use C5OH and considered that 3-MCC was an incidental finding in detecting 3-hydroxy-3-methylglutaric aciduria and beta-ketothiolase deficiency [13]. 

The classification of remethylation defects was contentious amongst committee members. They are detected using low methionine levels, and there was general agreement that it is more difficult to detect screened conditions through low as compared with high analyte levels. However, whilst some believed that screening sensitivity could still be assessed and monitored, others believed that screening sensitivity was uncertain and thus thought that target criteria were not met. Overall, they were considered to be target disorders by three out of six Australasian screening laboratories and incidental findings by the remainder. Remethylation disorders are listed as additional secondary conditions in the Californian screening panel.

As of February 2023, guanidinoacetate methyltransferase (GAMT) deficiency was only considered a target condition in the Melbourne screening laboratory. This is because the Melbourne laboratory and published methods use derivatised bloodspots, and the remainder of the Australasian laboratories have underivatized assays [22]. However, it is likely that this disorder can still be screened for after appropriate pilot studies [23]. Of note, this was one of just two core RUSP conditions not included in the Californian screening panel (i.e., GAMT deficiency and Pompe disease).

## 4. Discussion

A greater number of listed conditions is often considered to equate to ‘better’ screening, whereas it may be due to conditions being counted differently. Through a consensus-based process, we developed a standardised approach to counting target disorders in newborn bloodspot screening across Australia and New Zealand and demonstrated that screening panels were broadly similar to each other as well as to the Californian bloodspot panel. Despite advocacy claims of the Californian programme screening for more than 80 conditions, these were counted as just 33 target bloodspot disorders. This discrepancy largely appears to have arisen from the misinformed counting of non-core and secondary conditions. This includes several, such as SCAD deficiency and Duarte galactosaemia, that most NBS programs now take conscious steps to avoid reporting. This illustrates the need to temper the enthusiasm of advocacy groups with informed professional advice during the political lobbying process. 

Concern about ambiguity and a lack of uniformity in disorder counting on newborn screening panels is not new. This was highlighted as a national issue in the United States by Sweetman et al. nearly 20 years ago, shortly after the formation of the RUSP [24], and remains topical. For the past 2 years, the US Association of Public Health Laboratories (APHL) newborn screening condition counting task force has been working on a framework to achieve uniformity in how states count conditions [12]. In 2023, the task force recommended that secondary disorders be removed from the RUSP and other state programme disorder lists [25]. The HGSA NBS discussion was similarly complicated by the concepts of screening targets and incidental findings, for which the committee developed operational definitions. Whilst detecting incidental findings may (sometimes) be beneficial, by definition they have not been formally approved and are considered unlikely to meet Australian or New Zealand screening criteria. For example, all laboratories within our region detect cases of maternal B12 deficiency and consider there to be benefit in this. 

Gap analysis between the Australasian and Californian panels largely reflected differences in the jurisdictional approval of conditions and it remains the case that hemoglobinopathies and lysosomal storage disorders are not yet recommended in Australasia. Both Australia and New Zealand have well-defined processes for the consideration of newly screened disorders [1,16]. At the time of discussions, two conditions that were screened outside of Australasia (X-linked adrenoleukodystrophy and sickle cell disease) were under active consideration. Given the Australian government’s commitment to screening expansion, an increased number of disorders, including selected lysosomal storage disorders, are anticipated to be assessed for suitability to the Australian context over the next few years. As per the Wilson and Jungner criteria, part of the decision matrix should include the capacity of clinical services to deliver care to true positive cases and rapidly distinguish false positive and indeterminate cases [8]. In some cases, especially for rare scenarios, patients can be followed up for many years before it is clear whether they are affected or not [26]. The availability of cost-effective interventions, acceptable to society, should remain as a guiding ethical and policy principle. 

Differences between Australasian screening panels largely reflected deliberate decisions, based on local circumstances and the balance of screening benefits and harms as interpreted by different jurisdictions. As such, a greater number of differences were observed between the New Zealand and Australian panels than nationally between Australian panels. Tandem mass spectrometric analysis of acylcarnitines, amino acids, and other metabolites (MSMS screening) is a cost-effective method for detecting cases of rare, life-threatening inborn errors of metabolism. However, when this was introduced to NBS, individual disorders were not always considered in detail, as there was little information available about many of the rarer conditions [11]. Time and experience have increasingly led to the recognition of non-pathological cases of biochemical variation, prompting the removal of conditions from some screening panels. Both the Australian and New Zealand policy frameworks describe pathways for stopping screening for disorders, utilising the same criteria used for the consideration of new disorders [1,16]. For newly approved disorders, differences between Australian programmes reflected a varied timeframe in terms of being able to acquire the funding and resource needed to implement these. 

## 5. Conclusions

A harmonised approach to disorder counting is essential for valid programme comparison. An Australasian disorder list was developed in the context of Australian health funders wishing to harmonise and expand national screening panels. The approach used by the HGSA NBS committee may be useful to other groups who wish to compare screening panels. Further international standardisation of condition counting, as well as disorder definitions, would reduce ambiguity in programme comparison.

## Figures and Tables

**Table 1:** Definitions of target disorders and incidental findings.

Target disorders meet the following criteria:
Meets screening criteria for the jurisdiction; There is an intent to detect with maximum sensitivity and specificity; It is possible to determine sensitivity (which may not be high if appropriately balanced with specificity).
Incidental findings are those conditions which:
Have a marker metabolite the same as for a target condition but do not meet screening criteria; May or may not be detected by a programme, dependent on the markers and screening algorithm for the target condition; May not be able to be distinguished from the target condition in the screening laboratory without confirmatory follow-up testing.

**Table 2:** Comparison and gap analysis to “80 Californian conditions” which were counted as 33/35 core bloodspot conditions from the recommended uniform screening panel (RUSP) and the six Australasian newborn bloodspot programmes in February 2023.

Count	Condition	CA	NZ	NSW	VIC	QLD	SA	WA
	***Amino Acid Disorders***							
1	Argininosuccinic aciduria (ASA)	Y	Y	Y	Y	Y	Y	Y
2	Citrullinemia, type I (CIT)	Y	Y	Y	Y	Y	Y	Y
3	Classic phenylketonuria (PKU)	Y	Y	Y	Y	Y	Y	Y
4	Homocystinuria (HCY)	Y	Y	Y	Y	Y	Y	Y
5	Maple syrup urine disease (MSUD)	Y	Y	Y	Y	Y	Y	Y
6	Tyrosinemia, type I (TYR I)	Y	Y	N	N	N	N	N
	***Endocrine Disorders***							
7	Congenital adrenal hyperplasia (CAH)	Y	Y	Y	Y	Y	Y	Y
8	Primary congenital hypothyroidism (CH)	Y	Y	Y	Y	Y	Y	Y
	***Fatty Acid Oxidation Disorders***							
9	Carnitine uptake defect (CUD)	Y	N	Y	Y	Y	Y	Y
10	Long-chain L-3 hydroxyacyl-CoA dehydrogenase deficiency (LCHAD)	Y	Y	Y	Y	Y	Y	Y
11	Medium-chain acyl-CoA dehydrogenase deficiency (MCAD)	Y	Y	Y	Y	Y	Y	Y
12	Trifunctional protein deficiency (TFP)	Y	Y	Y	Y	Y	Y	Y
13	Very-long-chain acyl-CoA dehydrogenase deficiency (VLCAD)	Y	Y	Y	Y	Y	Y	Y
	***Hemoglobin Disorders***							
14	S, Beta-Thalassemia (Hb S/ßTh)	Y	N	N	N	N	N	N
15	S, C disease (Hb S/C)	Y	N	N	N	N	N	N
16	Sickle cell anemia (Hb SS)	Y	N	N	N	N	N	N
	***Lysosomal Storage Disorders***							
17	Mucopolysaccharidosis Type-I (MPS I)	Y	N	N	N	N	N	N
18	Mucopolysaccharidosis Type-II (MPS II)	N	N	N	N	N	N	N
19	Pompe (POMPE)	Y	N	N	N	N	N	N
	***Organic Acid Conditions***							
20	3-hydroxy-3-methylglutaric aciduria (HMG)	Y	N	Y	Y	Y	Y	Y
21	3-methylcrotonyl-CoA carboxylase deficiency (3-MCC)	Y	N	N	N	N	N	N
22	Beta-ketothiolase deficiency (BKT)	Y	N	Y	Y	Y	Y	Y
23	Glutaric acidemia, type I (GA-1)	Y	Y	Y	Y	Y	Y	Y
24	Holocarboxylase synthetase deficiency (MCD)	Y	Y	Y	Y	Y	Y	Y
25	Isovaleric acidemia (IVA)	Y	Y	Y	Y	Y	Y	Y
26	Methylmalonic acidemia (Cobalamin Disorders) (Cbl A,B)	Y	Y	Y	Y	Y	Y	Y
27	Methylmalonic acidemia (Methymalonyl-CoA Mutase Deficiency) (MUT)	Y	Y	Y	Y	Y	Y	Y
28	Propionic acidemia (PROP)	Y	Y	Y	Y	Y	Y	Y
	***Other Disorders***							
29	Adrenoleukodystrophy (ALD)	Y	N	N	N	N	N	N
30	Biotinidase deficiency (BIOT)	Y	Y	N	N	N	N	N
31	Classic galactosemia (GALT)	Y	Y	Y	N	Y	Y	Y
32	Cystic fibrosis (CF)	Y	Y	Y	Y	Y	Y	Y
33	Severe combined immunodeficiency (SCID)	Y	Y	Y	N	N	N	N
34	Spinal muscular atrophy (SMA)	Y	N	Y	N	N	N	N
35	Guanidinoacetate methyltransferase (GAMT) deficiency	N	N	N	Y	N	N	N

Green = target condition screened; yellow = screening incidental finding; red = screening stopped; pink = screening intended; blue = screening not approved. CA = California; NZ = New Zealand; NSW = New South Wales; VIC = Victoria; QLD = Queensland; SA = South Australia; WA = Western Australia.

## Data Availability

The original contributions presented in the study are included in the article, further inquiries can be directed to the corresponding authors.
